# Philanthropy patterns in major Australian performing arts organizations

**DOI:** 10.1007/s10997-022-09657-2

**Published:** 2023-01-12

**Authors:** Chiara Carolina Donelli, Ruth Rentschler, Simone Fanelli, Boram Lee

**Affiliations:** 1 Department of Management and Economics, University of Parma, street J. F. Kennedy, 43123 Parma, Italy; 2 Department of Management, Ca’ Foscari University of Venice, Venice, Italy; 3 UniSA Business, University of South Australia, Adelaide, Australia

**Keywords:** Philanthropy, Performing arts, Resource dependence, Resource-based view

## Abstract

Taking a management perspective in the field of philanthropy, this study examines 12 Australian major performing arts organizations over 19 years (2000–2018), which were identified as vulnerable and struggling with overreliance on public grants. Underpinned by theories that integrate understandings of external and internal resource management—resource dependence theory and the resource-based view—we uncover insights into what drives the increase in their philanthropic income. Using data from 228 annual reports and interviews, we present an original taxonomy that identifies organization-donor relationships and organizational efforts in nurturing philanthropy. We uncovered the interplays between donor engagement and positioning philanthropic staff in terms of organizational structure. Longitudinal financial and narrative data demonstrate that external resource management through donor engagement and internal resource management through organization structure emphasizing philanthropy have a significant impact on the growth of organizational philanthropic income.

**Supplementary Information:**

The online version contains supplementary material available at 10.1007/s10997-022-09657-2.

## Introduction

Governments worldwide have been calling on arts organizations to diversify their income for financial sustainability (e.g., see Radbourne & Watkins, 2015) and strategic usage of resources (Reficco et al., 2020) as part of a wider reform program of New Public Management (Pollitt & Bouckaert, 2011) covering both public and non-profit sectors. New Public Management theory is influenced by neoliberal notions of capitalism and interventions by the state, and it holds that revenue enhancement through philanthropy is essential in non-profit arts organizations (Alexander, 2018). Over the last 60 years, public financial support from government to the non-profit sector has fallen (Moldavanova & Goerdel, 2018; Weerawardena et al., 2010), particularly after the global financial crisis, which placed further pressure on non-profit organizations (NPOs) to compete for other funding sources (Cho & Gillespie, 2006; Carroll & Stater, 2009; Boddewyn & Buckley, 2017). Cultural organizations have developed a variety of responses to the pressure; increasing revenue from philanthropy is an important one (Cobb, 2002).

This study takes a management perspective on the notion of philanthropy, contributing to the literature for those public and non-profit organizations for which philanthropy is not the core activity. In the non-profit sector, PAOs are particularly vulnerable to resource scarcity, as postulated by Baumol and Bowen (1966), and tread a fine line between meeting financial sustainability needs and achieving their social and aesthetic mission (Fanelli et al., 2020). The increased competition for limited revenue sources has resulted in two different scenarios: (1) PAOs trying to increase production of goods and services with a more commercial orientation; and (2) PAOs relying more on philanthropic funding (Ecer et al., 2017). In order to ensure mission delivery and not fall back into commercialization of their activities (McCaskill & Harrington, 2017), PAOs have spent considerable time and money in accessing, acquiring or developing resources which can be strategic (Knockaert et al., 2013) in attracting private funds to realize artistic achievement (Labaronne, 2019). Philanthropy is thus an important source of revenue for the non-profit sector, both for ongoing activity and in times of crisis (Rubio-Arostegui & Villarroya, 2021), as it is seen to be less restrictive than government funding which usually connects with specific projects (Phillips & Jung, 2016).

However, while there is now greater understanding of philanthropy, there is less understanding of the relative importance of philanthropy vis-a-vis other factors that can drive organizations towards private income. One explanation for this is that scholars have used an ‘either/or’ approach to developing singular theoretical frameworks (e.g., Sandhu, 2013) to examine drivers of philanthropy. However, many PAOs have failed to provide effective results, emphasizing the need to focus on external as well as internal efficiency (Osborne et al., 2015).

Previous studies have used Resource Dependence Theory (RDT) (McGrath & Legoux, 2017) which focuses on external resources alone; or the Resource-Based View (RBV) (Schauerte et al., 2020) which examines the role of internal resources on institutional drivers (e.g., Pierce, 2000). While a singular framework may be suitable for examining linear relationships, it does not suit complex phenomena (Sandhu, 2013), such as the changing patterns of philanthropy in PAOs enacted over time. This article responds to the call to examine the complex interplays between ‘multiple determinants at multiple levels’ (Sandhu, 2013) by integrating theories on external and internal resource management—RDT and RBV—in PAOs in order to create an integrative framework of financial sustainability for NPOs through philanthropy. We argue that the use of ‘simple mechanisms’ of donor engagement and organizational structure have ‘profound consequences’ (Alexander, 2018, p. 33), in an increasingly neoliberal state. Through the use of the two theories, we analyze the interplays between the organizational structure in relation to philanthropy according to Mintzberg’s framework (1992); and the engagement of the organization with donors through stakeholder community relations based on Bowen et al.’s (2010) taxonomy. Referring to organization-donor relationships, we call transactional engagement a situation of one-way communication processes in which the organization ‘informs’ the target audience. We call transitional engagement a situation of two-way communication, where however the flow is asymmetrical with strong lines of communication.

The contribution of this article lies in the integration of the two theories: RDT and RBV. It uncovers uneven patterns of philanthropy. Generally, the shift is to transitional donor engagement rather than transactional, and there is low-level structural positioning shifting to the apex of the organization for better results. This type of shift has received little academic attention for non-profit organizations (Raffo et al., 2016). We argue that the shift occurs by showing that major PAOs have become more heteronymous over time as they take on certain characteristics of neoliberalism (Alexander, 2018), which are evident in changes in donor engagement and organizational structure. Heteronymous changes see PAOs becoming more managerialist in the manner in which they handle philanthropy and how they structure their organizations (Alexander, 2018). In this scenario, neoliberalism is brought about by resource dependencies (Alexander, 2018) and through coercive pressures and changing government requirements (e.g., Nugent, 1999). Integrating these different theories made it possible to develop an empirical framework which extends theory and can also be applied to interpreting neoliberal shifts in PAOs.

This study analyzes the major performing arts sector in Australia, relevant to other Western nations with a common law tradition whose PAOs are struggling in uncertain times. Philanthropic behavior is diverse and country-specific (Bekkers & Wiepking, 2011), and our paper is set in Australia as a country with a tradition of philanthropy, and a philanthropic potential comparable to other countries of the Commonwealth (Bekkers & Wiepking, 2011). Unlike American cultural organizations, which rely significantly on private sources of support (Toepler & Wyszomirski, 2012), Australian ones receive government support. However, as reported in the *Giving Australia Report* (2018), philanthropy in Australia is growing and expanding in ‘new (or recently revived) mechanisms’, with a growing emphasis on giving being ‘everyone’s business.’ Australian PAOs are also representative of a vulnerable non-profit sector, which is reliant on external resources and is undergoing transformation, as noted in reports commissioned by the Australian Government (e.g., Nugent, 1999; 2016).

The rest of this article is structured as follows. A brief review of the literature is followed by a focus on theories of RDT and RBV, and our research questions are outlined. The next sections describe methodology and findings. The conclusion notes limitations of the research and indications for future research.

## Literature review

The literature review first examines philanthropy, then external resource management, and internal resource management. At the end of each of the three sections, a research question is posed.

### Philanthropy

The term philanthropy derives from early English usage for e.g., ‘goodness,’ ‘benevolence’ and ‘goodwill towards fellow man’ (Sulek, 2010). The term has undergone significant change over time, in ways that illuminate both contemporary and academic understanding of it (Lindahl & Conley, 2002). Philanthropy and fundraising have been used interchangeably and the two concepts sometimes fall under the umbrella of ‘patronage’. Fundraising is a social exchange between the agency (cultural organization) expressing a need, and the prospect (the donor) who donates in exchange for tangible benefits (e.g., public recognition) or intangible benefits (e.g., self-actualization, personal satisfaction) (Radbourne & Watkins, 2015). Philanthropy has emerged more recently as a ‘new concept’ (Cobb, 2002), which has grown from purely transactional raising money to shared value co-creation, termed by Drucker ‘people development’ (Drucker, 1995, p.74).

Philanthropy is thus defined as the application of private means which can be money, time, goods or effort to meet public ends (Sulek, 2010; Harrow & Jung, 2011; Jung, 2015). Financial support is the consequence of building relationships and sharing values with external constituents (Voss & Voss, 2000), or developing a sense of shared organization ownership (Drucker, 1995), which has a strategic intent. Philanthropy is in fact underpinned by different interactions between potential donors and organizational strategy, including: (1) shared responsibility for development; (2) integration and alignment with the organization mission; (3) focus on fundraising as engagement; and (4) developing and sustaining strong donor relationships (Gibson, 2016). Different organizations wishing to strengthen their philanthropic income thus require different processes for their business model.

There is an extensive body of conceptual and empirical work on philanthropy from different perspectives. To date, the field has been dominated by motivational studies using an individual perspective (Bertacchini et al., 2011; Lindahl & Conley, 2002); and a corporate perspective (Selma et al., 2020; Webb, 2004). Other studies investigate how external factors can affect donation behavior, such as government support (Brooks, 1999; Borgonovi, 2006) and tax benefits (Donelli et al., 2022; Mulcahy, 1998; Pharoah, 2010). Shifting the focus from donors to recipient cultural organizations, studies on philanthropy have been undertaken mainly in relation to philanthropic foundations (Toepler, 2018), and venture philanthropy (Moody, 2008) for which raising funds is the core activity of their business model. In addition, most academic research into business support for the arts has been undertaken in the U.S., where fundraising is historically an important function of their management and focus (Bell, 2012). Although country specific studies with non-US perspectives are now appearing in the arts (Rubio-Arostegui & Villarroya, 2021), the field is still relatively new.

The Australian government commissioned reviews of Australian PAOs in 1999 and 2016 from Dr. Helen Nugent, who was tasked with inquiring into legislative frameworks, governance, funding, and structure of PAOs, with the aim of ‘secur[ing] the future,’ recommending diversifying income and ‘restructur[ing]’ in order to achieve a ‘viable sector’ and artistic ‘vibrancy’ (Nugent, 1999). Factors justifying using the Australian arts scene as appropriate for analysis for resource dependence in relation to philanthropy, include: (i) *a broad arts sector*: it contributed A$111.7 billion to Australia’s economy in 2018, or around 6.4% of GDP (Australia Council for the Arts, 2018); (ii) *limited diversification*: Australia tends to be governed according to New Public Management theory, and an increasingly neoliberal philosophy which has spread into the performing arts (Alexander, 2018; Pollitt & Bouckaert, 2011); Australia was traditionally characterized by a welfare state philosophy in the arts, although to a lesser extent than most advanced western countries (Gardiner-Garden, 2009); Australia has a cultural disposition toward privacy; and an arguably weak culture of philanthropy (Liffman, 2008).

Australian PAOs in their current form date from the mid-20th century, when they were set up with support mainly from the federal government’s arts funding and advisory body, the Australia Council for the Arts. PAOs in Australia follow the same processes as other Western countries influenced by New Public Management and neoliberal reforms: often set up as public entities, they have shifted over the last 20 years into non-profit status or a form of public-private organization (Kawashima, 1999). They have also restructured and changed approaches to donor engagement in order to meet neoliberal pressures (Alexander, 2018). Furthermore, government-commissioned reviews of major Australian PAOs (e.g., Nugent, 1999; 2016) identified opera, dance and theatre as the most vulnerable PAOs in the sector with a need for philanthropic income as a means to ‘strengthen,’ ‘stabilize and reposition’ the sector as part of wider ‘industry restructuring.’ Nonetheless, the 2016 report identified continuing strategic and structural challenges due to resistance to re-structuring (Nugent, 2016). Nugent argued that the ‘companies’ strategic roles [allocated] are unclear;’ their ‘financial dynamics [remain] unsustainable;’ and they have ‘inadequate staff resources to increase earned income’ (pp. 14–15). Despite this, there has been no study examining the effect (if any) of changes in government policy on the ability of major Australian PAOs to attract philanthropy.

Hence, our first research question is: *How has philanthropic income changed in major Australian performing arts organizations in the 21st century?*

### Donor engagement

Philanthropy and revenue diversification have been given less attention in traditional non-profit management research, so we looked to the classic management scholar, Drucker (1995) in examining philanthropy. We build on his work, which examined not only internal resource management but also external resource management. Following Drucker (1995), we examine two theories, RDT and RBV, as a foundation for our study. RDT is defined as the provision of external resources that have a unifying theory of power inequalities between stakeholders at the organizational level of analysis (Pfeffer & Salancik, 2003). In this theory, organizations pursue strategies to reduce uncertainty about organizational survival by controlling access to or use or possession of resources (McGrath & Legoux, 2017; Charreaux & Desbrières, 2001). Each source of income (McGrath & Legoux, 2017; Schauerte et al., 2020) has constraints and pressures which may impinge on mission achievement if not handled appropriately (Froelich, 1999). For PAOs, strategic resources include philanthropic income to fund activities for their mission (McCaskill & Harrington, 2017). Organizations pay attention to external stakeholders, such as donors, who are key to securing financial resources (Foster et al., 2009; Boddewyn & Buckley, 2017).

Establishing lasting relationships with external stakeholders such as donors, based upon mutual respect and trust, is a strategic way to manage the external environment in order to achieve organizational goals (Dunn, 2010; Cermak et al., 1994), through financial support that builds relationships and shares values (Voss & Voss, 2000). In the non-profit sector, establishing relationships with external stakeholders was previously understood as necessary to develop boards of directors which might be able to attract external resources who directly impact performance, thus reducing dependency on government (Callen et al., 2010; Romero-Merino & García-Rodriguez, 2016; Pfeffer & Salancik, 2003).

However, according to Herremans et al. (2016), organizations in fact engage stakeholders in different ways. They use transactional engagement to inform stakeholders so that the message is delivered to the recipient, creating organizational goodwill, reducing uncertainty. More inclusive public outreach (Moldavanova, 2016) aims to address the need of different stakeholders (Varbanova, 2013) rather than influence or change the organization. Transitional engagement entails two-way communication, which is more intense, and perceived as a dialogue between the organization and stakeholders (Bowen et al., 2010). While both Herremans et al. (2016), and Bowen et al. (2010) describe various engagement processes with stakeholders, neither study investigates how various engagement types can influence philanthropic income. Of course, donor engagement strategy is but one area of PAO external resource management, but it yields a sophisticated view of organizational culture and capabilities.

Hence, our second research question is: *How do different types of donor engagement influence philanthropic income in performing arts organizations?*

### Organizational structure

RBV focuses on factors internal to the organization which help to obtain resources and influence them to be responsive to contextual pressures (Schauerte et al., 2020). In 1984, Wernerfelt was the first to propose a shift in how organizations were analyzed, moving from a product-market focus to a resource-position focus, examining the link between an organization’s internal characteristics/resources and performance. To date, RBV is the dominant theoretical framework for understanding heterogenous organizational performance. Researchers in the last 20 years have identified the different types and combinations of tangible and intangible resources that allow organizations to attract resources (Zubac et al., 2010).

Organizational internal resources can be clustered conveniently into three groups: physical; human; and organizational capital (Smart & Wolfe, 2000). Organizational resources include firm planning, controlling and coordinating systems, as well as the relationship among employees and groups. In other words, organizational resources make up the organizational structure (Rose et al., 2010). The characteristics of internal organizational resources in all three types of resource are decisive for creating organization success and performance (Lado et al., 2006), while organizational culture and capabilities can influence the way in which these resources are attracted, managed and used in business processes (Barney, 1986; Jones et al., 2005). Teece et al. (1997, p. 516) define organizational capabilities as the “ability to integrate, build and reconfigure internal competences”. However, among the various internal resources, several researchers argue that organizational structure is particularly significant. According to Okumus (2003), the way in which organizational structure is defined determines not only the organization’s ability to implement strategy, but also to achieve a competitive advantage. Pertusa-Ortega et al. (2010, p. 1283) state that “RBV may be more appropriate to analyze the relationship between organizational structure and competitive strategy”. The term ‘organizational structure’ refers to the formal configuration between individuals and groups regarding allocation of tasks, responsibilities, and authority (Greenberg, 2011). Organizational structure is a strategically relevant resource to enable an organization to exploit external opportunities, such as philanthropic giving (Chatzoglou et al., 2018; Miles et al., 2011). In addition to that, although RBV has become dominant within the field of management, it has had surprisingly little influence within organization theory (Davis & DeWitt, 2021).

In this scenario, RBV, which emphasizes internal attributes, allows scholars to reframe the relationship between strategy (Lindley & Wheeler, 2000) and structure by analyzing the organizational structure as a valuable resource and a source of competitive advantage (Pertusa-Ortega et al., 2010). Although RBV has been developed in the for-profit sector, the theory is also relevant to non-profit organizations and the social economy (Akingbola, 2013) as these organizations need to build capabilities to attain organizational outcomes such as funding and reputation (Arya & Lin, 2007).

RBV can thus identify PAO resource deficiencies caused by external shocks, prompt internal organizational restructuring and the development of new role incumbents to meet changing organizational needs (Raffo et al., 2016; Sandhu & Kulik, 2019). Restructuring of organizational roles inevitably impacts philanthropy, in terms of which part of the organizational structure to position it in and which tasks and responsibilities to assign. They are a clear expression of the strategic orientation of the organization in achieving its goal of philanthropic income (Miles et al., 2011; Mintzberg, 1992). Mintzberg suggests that organizations consist of five main parts: the strategic apex (top management), middle line (middle- and lower-level management), operative core (workers who actually carry out the organization’s tasks), technostructure (analysts such as engineers, accountants, planners, researchers, and human resource managers), and support staff (people who provide indirect services). Mintzberg (1992) argues that organizational structure influences strategy and performance; we thus discuss the position of philanthropic staff in the organizational structure following Mintzberg’s framework (1992).

Hence, our third research question is: *How does the organizational structure for philanthropy influence philanthropic income of performing arts organizations?*

## Method

### Study setting

We undertook a multi-perspectival study, using data from 12 PAOs (see Table 1), and obtained robust theoretical insights from hard and soft data respectively.

**Table 1:** Characteristics of PAOs.

Established	Organization	Location State	Size*
1952	West Australian Ballet (WAB)	Perth (WA)	Small
1953	Melbourne Theatre Company (MTC)	Melbourne (VIC)	Large
1956	Opera Australia (OA)	Sydney (NSW)	Large
1960	Queensland Ballet (QB)	Brisbane (QLD)	Medium
1962	The Australian Ballet (TAB)	Melbourne (VIC)	Large
1970	Queensland Theatre Company (QTC)	Brisbane (QLD)	Medium
1972	State Theatre Company of SA (STCSA)	Adelaide (SA)	Small
1976	State Opera of South Australia (SOSA)	Adelaide (SA)	Small
1978	Sydney Theatre Company (STC)	Sydney (NSW)	Large
1981	Opera Queensland (OQ)	Brisbane (QLD)	Small
1989	Bangarra Dance Theatre (BDT)	Sydney (NSW)	Small
1991	Black Swan Theatre Company (BSTC)	Perth (WA)	Small

Notes: * Companies are treated as “large” if their turnover exceeded A$15m, “medium” for companies with a turnover between A$8m and A$15m, and “small”, less than A$8m in the year 2017

PAOs studied entailed a sub-population of 12 within the population of [then] 28 Australian major PAOs recognized for their national leadership and artistic excellence by the Australia Council for the Arts. The PAOs are limited to the art forms of opera, dance and theatre, the most vulnerable sectors, most negatively affected by the introduction of large-scale commercial musicals, festivals and spectaculars (Recommendation 3.2, Nugent, 1999). Further, they were chosen to ensure geographic spread, representing six Australian federated states (administrative divisions: New South Wales, Queensland, South Australia, Tasmania, Victoria, and Western Australia), except for Tasmania, where no PAO was present in the genre (opera, dance or theatre). Three out of 12 performing arts organizations in our sample, namely, Opera Australia, Queensland Theatre and State Opera of South Australia were—at the time of the study—statutory authorities (i.e., government-owned public corporations overseen by various state government departments). Some of these organizations are transitioning into independent governance structures e.g., companies limited by guarantee. PAOs are controlled by state regulations, and the levels of funding they receive from the state vary from 22 to 45%, according to their 2018 annual reports, with the expectation for them to self-generate income.

### Data collection

Data were collected in two stages with the aim of triangulating findings to reduce the impact of potential bias (Bowen et al., 2010).

#### Phase 1: Annual reports & documents

Documents were collected, including government commissioned reports, web sites of PAOs, and 228 annual reports from 12 PAOs from a period of 19 years (2000–2018). These documents provided a useful means of tracing change and provided rich descriptions of phenomena. Data on different revenue sources were extracted from financial statements. Income sources were identified as: public grants (including federal, state and local); sponsorship; philanthropy; and earned income (e.g., revenue from box office, rent and cafés). All financial reports were prepared in accordance with Australian Accounting Standards adopted by the Australian Accounting Standards Board and the Australian Charities and Not-for-profits Commission. Although annual reports are variable in content and form (Christensen & Mohr, 2003), they provide similar information, are produced each year and reflect organizational mission and values, as well as financial data (Michalisin, 2001; Rentschler et al., 2019).

#### Phase 2: Interviews

Methodologically, the study paired evidence found in financial statements in annual reports (ARs), with content analysis of the chair, CEO and philanthropy reports. Philanthropy is not the core business of PAOs, and the ARs show that the role played by philanthropy differs widely. Some organizations develop specific philanthropy reports, where they make explicit their vision of philanthropy and strategic direction, while others do not mention philanthropy either in ARs or in the CEO or executive director reports, and only acknowledge major donors. This difference in terms of attention and space in the different ARs inspired the authors to investigate further. In the past, research speculated on resource dependence by using secondary data (Rentschler, 2015), and here we used interviews (Judge & Zeithaml, 1992; Rentschler, 2015) as secondary data as well as direct evidence from inside the organization.

We contacted the 12 PAOs to interview key informants within each one, in order to obtain insights to triangulate the study. We interviewed the key person responsible for philanthropy, who sometimes sat at the organization strategic apex; middle line; or operating core in the organization structure. Depending on the organizational structure, the informants were board directors, CEOs, philanthropy managers, marketing managers, philanthropy coordinators, at the executive, middle and operational levels. On the basis of the interviews, we categorized each organization as to whether it adopted transitional or transactional approaches to donor engagement.

The categories used, the classifications made, and the different levels of coding are discussed in the next section, in accordance with Gioia et al.’s (2013) approach. The profile of interviewees is summarized in Table 2. Semi-structured interviews were conducted based on broad, open questions around key themes which emerged from the literature (Lapan, 2003): donor engagement; organizational structure; and philanthropic income.

**Table 2:** Characteristics of interview participants

N	Alias	Gender	Age range	PAO	Position		
					**Level**	**Summary**	**No. Phil. Staff (FTE)**
			**Executive and operational staff**				
1	Kent	Male	46–55	Dance	Strategic apex	Philanthropy director	9.5
2	Dan	Female	36–45	Theatre	Strategic apex	Philanthropy director	6
3	Lisa	Female	46–55	Opera	Strategic apex	Philanthropy director	5.5
4	Noemi	Female	46–55	Dance	Strategic apex	Development manager	3
5	Andrea	Female	36–45	Theatre	Strategic apex	Philanthropy manager	1.5
6	Chloe	Female	46–55	Theatre	Strategic apex	Philanthropy manager	1
7	Jane	Female	36–45	Theatre	Middle line	Development manager	4.5
8	Toni	Male	26–35	Dance	Middle line	Philanthropy manager	3
11	Sally	Female	66+	Opera	Operating Core	Philanthropy role	0.5
12	Luisa	Female	66+	Opera	Strategic apex	Board member	0.6
13	Deborah	Female	46–55	Dance	Strategic apex	CEO	0.8

### Data analysis

Analysis started by comparing different revenue sources used by the 12 PAOs and their changes over time. We then examined the external resource dependence of the 12 PAOs by examining annual report content and interviews with reference to engagement. Combining engagement with donors and organizational structure offers complementary views of resource dependence in PAOs.

We analyzed data as we collected it, making multiple iterations between data and emerging theoretical arguments (Sandhu & Kulik, 2019). ARs and interview data were analyzed in three stages, following previous qualitative studies (Sandhu & Kulik, 2019; Pratt et al., 2006):

(i) categorizing raw data into first order themes; first order themes included quotes extracted from interview transcriptions which were categorized by two independent researchers. In practice the researchers read the interview transcripts and started categorizing the raw data into ‘informant-centric first-order empirical themes’ (Sandhu & Kulik, 2019). Narrative data were coded using NVivo 12.
(ii) abstracting and consolidating themes into second-order conceptual categories; this included the coding process, consolidating empirical themes into higher-level conceptual categories which helped the researchers to better identify comparable patterns and actions implemented from different organizations.
(iii) aggregating conceptual categories into theoretical dimensions which are linked to the theory. After the second-order conceptual categories were generated, we categorized codes and how they fit together. At this point we explored the theoretical explanations for the categorization and aggregation we made among second-order categories. We based our approach on previous studies: Bowen et al. (2010) and Radbourne and Watkins (2015) on philanthropic engagement and Mintzberg (1992), on organizational structure.

We adopted Gioia et al.’s (2013) systematic approach to coding to develop categories related to previous studies in the field regarding the two themes analyzed: engagement and structure. Hrebiniak and Joyce (1985) argue that deterministic and non-deterministic perspectives both need to be used to understand organizational behavior. We used them both. Organizational structure and engagement were retrospectively classified within the formation and evaluation phases of studying the philanthropy categories. We based our assessments on outsider perspectives through annual reports and insider perspectives through interviews, building on previous studies which critiqued survey behavior (Judge & Zeithaml, 1992).

## Findings

In order to examine patterns of philanthropy taking place over time in PAOs, and to answer the three research questions, we analyzed the percentage of different revenue types; management of external resources through donor engagement; and management of internal resources through organization structure in relation to philanthropy.

### Philanthropic income

**Fig. 1:**
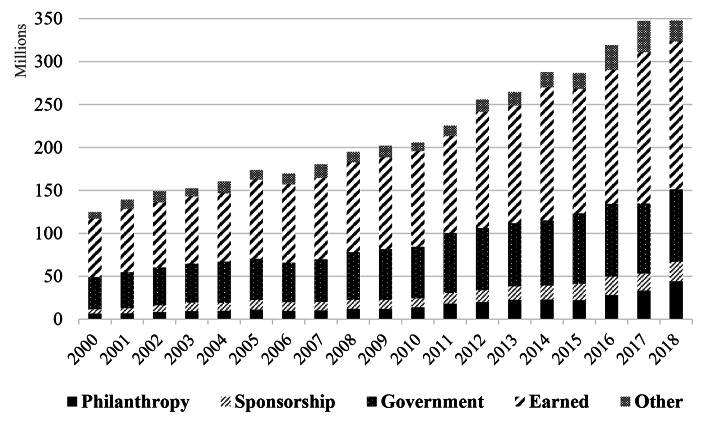
Revenue composition of all PAOs (2000–2018)

In order to answer our first research question, how philanthropic income changed in major Australian PAOs in the 21st century, different revenue sources were extracted from income statements in the financial reports of the 12 PAOs over 19 years (2000–2018). Financial performance is assumed to be a partial outcome of emergent philanthropic strategy of the PAOs examined. In order to determine whether PAOs were able to achieve financial viability, required by the New Public Management approach, we examined diversification of PAO revenue portfolios (philanthropy versus other income). Figure 1 illustrates that philanthropic income rose constantly over time with an average annual growth rate of 7.3%, while proportionally public grants marginally decreased over time. Sponsorship shows a gradual increase, but at a much lower level than philanthropic income, with an average annual growth rate of 1.8%.

In 2018, the PAOs in our study collected around A$67 million from private support, of which 66% was from philanthropy (A$44,130,012) and 34% from sponsorship (A$22,869,751). Private support received by the PAOs, on average, represented 19.1% of their total revenue (11.7% for donations and 7.4% for sponsorship). So, unlike similar organizations in the US, Australian PAOs did not rely significantly on private support. In 2000, private support for PAOs amounted to only around A$12 million, of which 55% was from philanthropy (A$6,452,829) and 45% from sponsorship (A$5,352,036), together accounting for about 10% of their total revenue (4.0% for donations and 6.1% for sponsorship). Overall, comparing 2000 to 2018, revenue increased by a factor of 2.8, but philanthropic income increased by a factor of 6.8.

The overall trend is shown in Fig. 1, while Appendix 1 shows for each individual PAO the dollar income from 2000 to 2018. It shows variations between different organizations in terms of revenue diversification. Our data show the overall trend towards revenue diversification, reflecting the ongoing reduction of public funding in the arts. This is observed around the world in the cultural sector due to the spread of neoliberalism. In Australia, the conservative coalition government (1996–2007) substantially restructured the funding system in the arts, requiring PAOs to be more business-oriented and to compete for funding (Power, 2022). This trend is also reflected in the main objectives PAOs set for their strategy, including public funding dependency and increase in the amount of revenue from philanthropy. This is illustrated in one of our cases:

*We continued to maintain our government dependency below 45% and our combined corporate partnerships and philanthropic support remained stable.* (CEO Report, AR, Queensland Dance Company, 2017).

**Table 3:** Average Revenue of PAOs in A$ and % (2000–2018)

	Philanthropy			Average (2000–2018)					
Organisation	2000	2018	*SD*	Philanthropy	Sponsorship	Public Grant	Earned	Other	Total
West Australian Ballet	81,997	1,316,774	886,799	675,538	912,707	3,419,754	1,958,672	103,407	7,070,078
%	3.80	11.70	5.64	7.12	13.41	48.50	29.53	1.45	100.00
Melbourne Theatre Company	226,889	2,196,526	977,262	890,630	979,733	2,214,050	11,286,950	3,848,410	19,219,774
%	2.57	7.11	2.97	3.96	4.66	12.09	59.44	19.86	100.00
Opera Australia	3,832,510	10,460,600	1,975,515	4,563,195	3,042,130	23,429,795	45,169,116	2,579,198	78,783,434
%	7.83	9.07	1.45	5.78	3.85	30.43	56.87	3.06	100.00
Queensland Ballet	64,694	4,805,315	1,774,818	1,167,879	867,080	2,595,611	2,318,558	536,175	7,485,302
%	2.55	22.95	9.54	8.94	7.67	44.93	31.95	6.51	100.00
The Australian Ballet	1,648,220	15,773,131	3,755,378	5,202,222	2,738,666	9,021,599	22,912,909	4,546,034	44,421,430
%	6.51	20.66	4.24	10.58	6.19	19.96	51.96	11.31	100.00
Queensland Theatre Company	51,000	1,697,000	393,955	260,192	528,656	4,415,950	3,221,789	316,368	8,742,956
%	1.08	12.10	2.70	2.35	5.87	51.85	36.31	3.62	100.00
State Theatre Company of SA	112,000	277,500	81,455	234,447	234,447	2,622,000	1,822,842	485,737	5,399,474
%	2.91	3.38	0.95	4.34	4.34	49.46	33.12	8.74	100.00
State Opera of South Australia	80,000	142,000	119,717	170,158	257,316	2,729,526	1,687,526	142,947	4,987,474
%	2.17	3.34	1.92	3.44	4.56	56.35	32.63	3.01	100.00
Sydney Theatre Company	0	4,323,377	1,297,109	2,554,891	1,791,242	3,626,525	19,476,570	2,184,100	29,633,327
%	0.00	9.87	4.92	8.62	5.86	11.87	67.39	6.26	100.00
Opera Queensland	66,964	422,944	143,754	217,065	507,531	3,046,197	1,853,661	242,555	5,867,009
%	1.35	5.80	2.15	3.55	8.78	52.00	31.36	4.32	100.00
Bangarra Dance Theatre	110,853	1,759,210	532,668	467,801	605,763	1,834,405	1,294,045	508,185	4,710,198
%	5.89	18.95	5.39	7.52	16.00	38.54	28.52	9.43	100.00
Black Swan Theatre Company	177,702	955,635	401,263	382,617	581,744	1,764,616	1,172,587	97,293	3,998,856
%	11.50	15.44	5.61	8.25	13.74	48.81	26.82	2.38	100.00
**Total**	537,736	3,677,501	2,203,356	1,398,886	1,087,251	5,060,002	9,514,602	1,299,201	18,359,943
	4.01	11.70	5.15	6.20	7.91	38.73	40.49	6.66	100.00

As shown in Table 3, philanthropic income varies widely between PAOs, with significant differences over the period. For example, Queensland Theatre Company, on average, obtained 2.4% from philanthropic income over 19 years, while The Australian Ballet obtained philanthropic income of more than 10%. As Table 3 shows, PAOs, such as Opera Australia and The Australian Ballet, attract large donations in dollar terms in the period analyzed; while others, such as State Opera of South Australia, attract on average A$170,158 over 19 years, which is 3.4% of their total revenue, and remain heavily dependent on public grants (56.4%). Bangarra Dance Theatre, which is an Indigenous Australian contemporary dance company, on average generated 16% of its revenue through sponsorship during 2000–2018, which is about twice the proportion of the revenue obtained from philanthropic income.

The standard deviation was calculated on both dollar and percentage terms of philanthropy income for each PAO from 2000 to 2018. In terms of percentage, the standard deviation of State Theatre Company of SA of 0.95 indicates little variance over time with almost no change from 2000 (2.9%) to 2018 (3.4%), while the standard deviation of Queensland Ballet of 9.5% demonstrates wide variation over time, as shown in the increase of the philanthropy income in 2018 (23%) in terms of percentage compared to 2000 (2.6%). In the following section, we explore the effect of donor engagement and organizational structure on philanthropy income based on our interview data.

### Donor engagement

In order to answer our second research question on how different types of donor engagement influence philanthropic income in PAOs, we examined engagement with donors in annual reports and interviews. Engagement with donors emerged as a differentiating factor in managing external resources for raising philanthropic income for PAOs. We observe a series of themes linked to the concept of engagement. A CEO of a dance company interviewed, sitting at the apex of the organization, highlighted the importance of donor engagement as a strategic relational activity:

*We are well connected, that has to be said. We work very, very, very hard. We don’t ask anyone [for a donation] until we know they love the art form. Building the relationship is so important.* (Deborah, CEO, Dance Company)

**Fig. 2:**
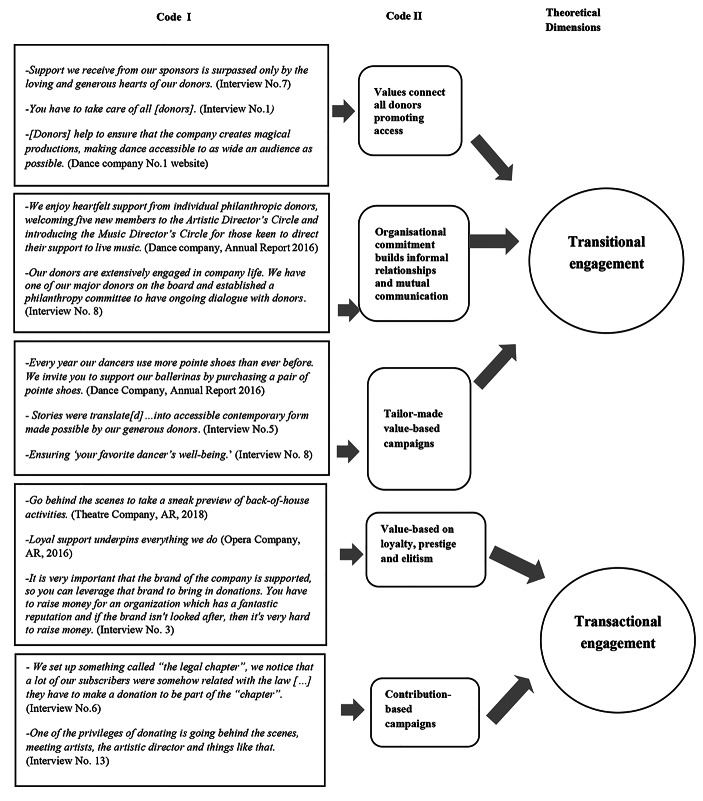
Donor engagement categories

However, varying forms of engagement can be identified in different philanthropic campaigns and include individual giving, annual giving, giving circles, legacies, and specific individual projects. For example, giving circles, or groups of donors who pool donations and decide what to finance, have become prominent in Australia over the last decade. As a form of donating, giving circles differ from traditional forms in that they involve the direct engagement and democratization of donors inside the organization (Boyd & Patridge, 2017). As shown in Fig. 2, according to Gioia et al.’s (2013) systematic approach to coding, from interview quotes we developed categories. Then, we grouped these categories into theoretical dimensions: the different levels of engagement, following previous RDT studies (Bowen et al., 2010), as either transitional or transactional.

*Transitional engagement* is present in PAOs that associate donors with ‘family,’ ‘friends’ or ‘community,’ aligning their own values with organization values. Donors play the role of co-creator, which is associated with being an investor in the future of the organization, or the sector in general.

Differentiated campaigns reflect varying strategies and commitment to donors of the PAOs examined. All donations are valued by organizations which engage closely with potential donors and range from ‘buy[ing] a pair of pointe shoes;’ and ‘one costume for a child performing;’ to ensuring ‘your favorite dancer’s well-being.’ Engagement values were expressed via visionary goals, for example, creating a future for generations to come; ‘being part of a family;’ ‘chang[ing] peoples’ lives;’ and ‘invest[ing] in the future of dancers and the entire country, or identifying a ‘number of unique ways you can become part of the family’ (Queensland Ballet, web site, 2017).

*Transactional engagement*, on the other hand, is associated with PAO need for cold, hard cash, with no mention of donors sharing PAO values. There in fact tends to be a lower level of engagement when value is linked to the prestige of belonging to a closed group of donors or supporters for the ongoing vitality of the brand. The goals of transactional engagement reflect the need for money and donations to support operational activities or build prestigious top-class performance. The level of engagement varies from tangible acknowledgement of the donation to being part of a ‘group of friends,’ ‘special circle’ or becoming an ‘ambassador’ of the organization. Donations are acknowledged, but mainly attention is given to sizeable endowments or gifts; high-profile donors are recognized by the CEO in the annual report. The value creation process is mainly a prerogative of the PAO and donors take part by providing funds. PAO strategy targets specific types of donor rather than specific values.

### Organizational structure for philanthropy

**Fig. 3:**
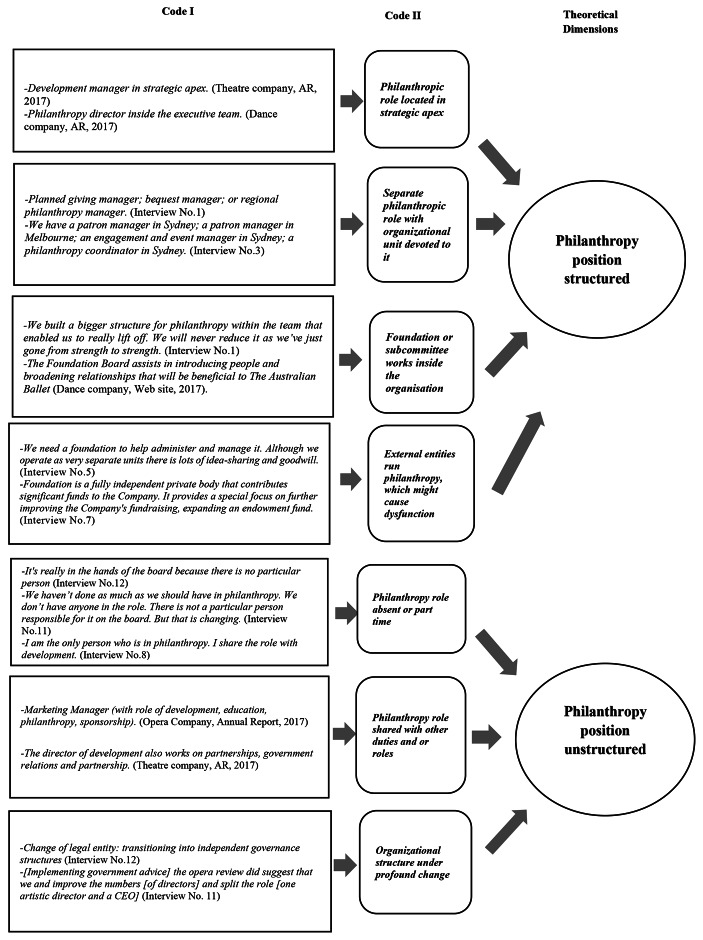
Organizational structure categories

In order to answer our third research question, how organizational structure influences resource acquisition of PAOs, we examined organizational structure of philanthropic roles in PAOs. Resource acquisition is an integral part of neoliberal organizational thinking, influencing key decisions and activities of role incumbents (Alexander, 2018; Radbourne & Watkins, 2015). Using an RBV perspective, we thus studied organizational structure as a valuable resource for organizational pursuit of strategy with the aim of growing philanthropy. RBV in fact emerges as an alternative explanation of performance differences between organizations in the strategic management literature (Mahoney, 2005), and was thus used in this study to investigate the difference in the ability of PAOs to attract donations. The organizational structure was examined with reference to philanthropy at the strategic apex (e.g., philanthropy role in the board of directors and executives; philanthropy director), middle line (e.g., philanthropy manager) and operational roles (e.g., philanthropy coordinator) in PAOs. Each position helped to provide a picture of the importance given to philanthropy in the PAO. Including people with specific tasks related to philanthropy in the strategic apex is a clear indication that the PAO considers philanthropy as a key element of strategy and resource acquisition (McNulty et al., 2011). At the other extreme, PAOs where philanthropy is only an operational role clearly consider it a marginal, sporadic and non-strategic activity. Finally, the presence of managerial roles in the intermediate line and directly connected to strategic apex show the PAO recognizes the importance of philanthropy for success, although it may be less strategically significant than other issues. Figure 3 shows, as we have categorized AR quotes and interviews into the different levels, according to Gioia et al. (2013).

All PAOs except for one have at least one philanthropy role, either full-time or part-time, in their organizational structure. Looking at how and at what level philanthropy is embedded into organizational structure shows that PAOs have invested differently, and that some PAOs have challenges to address to ensure that they continue to move forward. One philanthropy manager we interviewed at the strategic apex of the organization described the multiple challenges of organizational structure and donor engagement in order to succeed in raising funds:

*Our challenges were making sure we don’t stay top heavy and the money we needed, the small staff, and the speed at which we needed the money. That built the foundation.* (Chloe, Philanthropy Manager, Theatre Company)

Table 2 shows that there are PAOs which have several philanthropic staff and appoint them into higher positions such as planned giving manager, bequest manager, or regional philanthropy manager. This was the case especially if their ‘level of ambition’ was to put their PAO ‘on the world stage’. Managers with philanthropy duties, either philanthropic director or development director, sit on the leadership team at the strategic apex and implement strategies together with the artistic and executive directors, to ensure goals are co-created, shared and realizable. PAOs with these organizational features recognize the strategic role of philanthropy, assigning it rather than to technostructure or support staff, to a line function hierarchically relating directly to the strategic apex. In some cases, a donor committee or a foundation board works alongside the strategic apex. Generally, there is a board sub-committee, comprising ‘volunteer community leaders’ including philanthropists, which has the task of introducing people and ‘broadening relationships’ that are beneficial to the organization, thus ensuring constant exchange between donors and PAO.

Other PAOs have opted to place philanthropic staff in the middle or operational line of the organizational structure and to perform other roles as well, such as development manager. The leadership role, sitting on the executive team, does not necessarily work full-time on philanthropy. In some cases, executive directors spend part of their time on philanthropy and are supported by a coordinator. A foundation or external is sometimes present to manage funds received and ensure flexibility in decision-making (i.e., for statutory authorities). Here, philanthropy is at the strategic apex and donor engagement is prestigious:

*The MTC Foundation will ensure MTC remains an iconic Melbourne institution commissioning and developing new works in our state-of-the-art venue* (Melbourne Theatre Company, Annual Report 2016, Foundation Chair Report, p. 5).

Sometimes achievement is limited by a lack of leadership thinking. One philanthropy manager said: ‘[in this state] individual philanthropic giving is less mature’ Others stated that in their PAO, philanthropy is in the operational core of the organization, without strategic influence, sometimes with the position remaining vacant for a long time:

*I am the only person who is in philanthropy. I share the role with development. The philanthropy role had lapsed for two or three years before I came here. Previous incumbents were sometimes successful and sometimes not.* (Toni, Philanthropy Manager, Dance Company)

In other cases, PAOs ‘don’t see the benefits of having a foundation,’ limiting their philanthropic income opportunities, as one philanthropy manager with 1.5 staff in a small company of 15 people stated. The importance of structure to strategy was candidly admitted by another interviewee from a dance company experimenting with new ways of obtaining philanthropic dollars. This philanthropy manager explained that philanthropy was repositioned in the structure in order to engage better with donors, bringing greater financial rewards:

*We looked at the structure of our development team and saw big potential to grow philanthropic income, we noticed a real stagnation in support. … So, we put our effort into growing income from trusts and foundations and individual donors. … when we redeveloped our strategic plan. We became a lot better at explaining why we exist.* (Andrea, Philanthropy manager, Theatre Company)

There are a limited number of PAOs for which philanthropy is addressed together with other functions, such as marketing and communications, meaning that philanthropy duties are not central in the organization. The philanthropy tasks are either assigned to mid-line or operational staff or were not well defined. The position of philanthropy coordinator was present in two PAOs out of three, but in one was vacant for a year. In other PAOs philanthropy was overseen by a single board director giving his or her time on a voluntary basis.

## Donor engagement, organizational structure, and philanthropy

Performing arts organizations have changed in relation to donor engagement and organizational structure, which reflects the growing impact of neoliberalism (Alexander, 2018). PAOs previously relied on government income but today favor more business-centered approaches to donor engagement and organizational structure. Resource dependence has changed with the state putting pressure on PAOs to adopt these practices (Nugent, 1999). However, patterns of change are heteronymous rather than homogenous, with some PAOs reluctantly accepting the legitimacy of neoliberal ideology. The following discussion relates the findings to engagement with findings on structure, and clusters them into four categories in order to disentangle the effect of different donor engagement approaches and organizational structures on philanthropic income. The four clusters result from the intersection of the RBV (Labaronne, 2019) and RDT linking both to philanthropy. According to RDT, not only board members (Pfeffer & Slancik, 1978) but also the different types of engagement, acknowledge the needs of multiple stakeholders, an important factor of organizational success (Moldavanova, 2016). Indeed, the organization which focused primarily on resourceful donors had greater immediate organizational success. DiMaggio and Mukhtar (2004) find that an elitist approach is rife in the performing arts sector, which however cause less stability in terms of long-term sustainability and ability to attract resources.

**Fig. 4:**
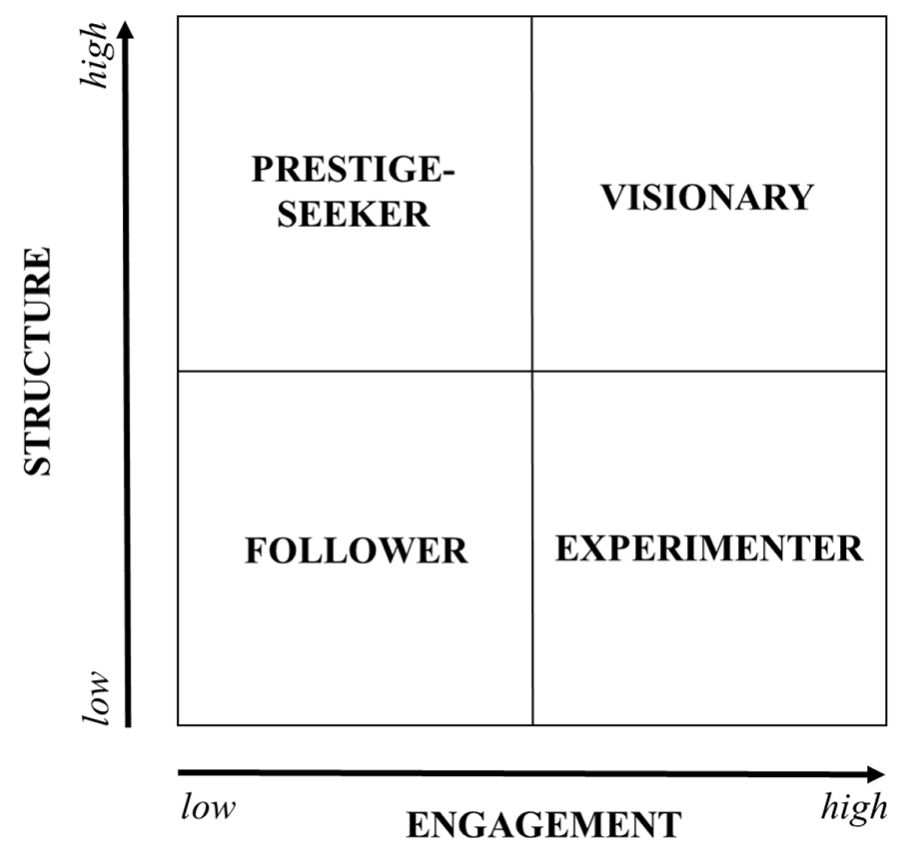
The relationship between philanthropic income trend, donor engagement and organizational structure

Figure 4 shows the complex relationships between philanthropic income trend, donor engagement and organizational structure. The categories are named *visionary* (transitional engagement and philanthropic formalization in the organizational structure at the strategic apex), *experimenter* (transitional engagement and unstructured position of philanthropy), *prestige-seeker* (philanthropic formalization in the organizational structure at the strategic apex with differentiated positions for philanthropy but transactional engagement) and *follower* (unstructured position of philanthropy and transactional engagement).

**Fig. 5:**
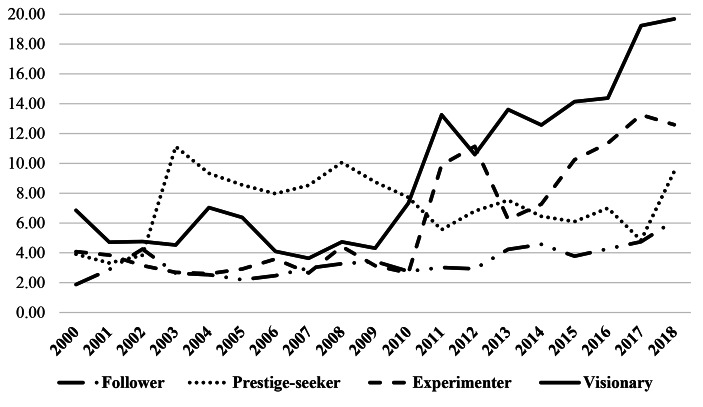
Philanthropic income (%) by category (2000-2018)

Each category shows different philanthropic income trends over the time period 2000–2018 as represented in Fig. 5. This categorization may not be fixed; it may shift over time as PAOs change their approach to philanthropic engagement, thus changing CEO, philanthropy manager or governance or management structure.

### Visionary

This group of PAOs engages with philanthropy as part of their core strategic vision and revenue portfolio. In practice, philanthropy has a clear impact on the strategic vision and business model of the whole organization. Revenue from donations (and the private sector in general) is a high percentage of annual income over the years examined, demonstrating active response to the Nugent (1999) report. There is a well-established, dedicated leadership team and organizational structure to support philanthropy strategy. The strategy is formulated at the highest level of the organization, with board direction, CEO and artistic director leadership, and philanthropy director guidance to ensure that goals are co-created, shared and realizable. In one PAO, there is a specialized philanthropic role at the strategic apex, whose position in the organizational structure placed her in a key role to influence philanthropic income while engaging with donors.

*I am the specialized person looking after philanthropy, in the executive team. Our strategy is embedded in our program. [The role] is about engagement. But engagement is dependent on whether the philanthropy person is full time or part time which affects the hours they can invest in building relationships.* (Chloe, Philanthropy manager, Theater Company)

Tailor-made programs and high-level donor engagement allow donors to create value for individuals, the organization and society. These PAOs, including their artists and artistic directors, engage with donors directly and/or through online media, creating value by telling a compelling story.

This group shows an ongoing upward trend. Every PAO obtains more than 15% of their income in donations. In all three cases, the proportion of public subsidy is below 50%, the median being 22%. Starting from a relatively high percentage of donations in 2001 (6%), these PAOs show a constant upward trend in donations (+ 157% in absolute value). There was a crucial turning point after the global financial crisis in 2009. The year 2018 illustrates the highest percentage of donations (about 21%) and the highest percentage of sponsorship. No crowding-in effect was observed as the average annual percentage change of public grants for these three PAOs was less than 10% while the annual percentage change for donations ranged from 20.5 to 43.7%.

### Experimenter

PAOs in this group experiment with different possibilities of structuring their organization in order to maximize philanthropic strategy and to engage and monitor donor needs. Creating or implementing a foundation is a claimed to be priority to maximize long-term sustainability; but it is yet to be realized. PAOs in this group have high-level engagement with donors. The outcome can be a high percentage of revenue from philanthropy, with spikes in conjunction with special campaigns (e.g., capital campaigns; anniversaries of the foundation establishment) or sporadic bequests, but revenue has not been consistent over the past decade, as philanthropy is not sufficiently embedded in the organization. The group provides evidence of PAOs innovating in order to develop revenue from donations while engaging with donors and telling a compelling story, providing a strong income source, thus ensuring financial viability and lower dependency on public grants. This group shows a fluctuating trend. In 2017, they received a high level of donations (32.8%, with a median of 4.3% and high standard deviation). However, they also register high levels of public revenue (about 40%). Moreover, their historic performance shows high revenue fluctuations from philanthropy over the years with spikes and sudden decreases. Philanthropy moves from a mean of 4% of donations in 2001 to 25.7% in 2018, including a drop in 2013 and 2014 of around 5% both years.

Some PAOs have a foundation which manages fundraising projects via a “fully independent private body” (Theatre Company 2016, Annual Report, p.8), which works in parallel, not within, the organizational structure, potentially leading to governance dysfunction and missed opportunities. On the other hand, interviews with PAOs whose philanthropic income was rising showed this could be a result of organizational restructuring (creating a role in the strategic apex) which boosted donor engagement:

*A few years ago, the Friends moved under our [the organization] wing. They are now part of the machine. We believe that the organization should be really close and connected to the true dance fan.* (Toni, Philanthropy manager, Dance Company)

### Prestige seeker

The group, which showed a downward trend over time in philanthropic income, includes PAOs which have high organizational attention towards philanthropy, including a philanthropy director positioned at the strategic apex. The board was often an early adopter in the field of fundraising, but the strategic direction is mainly toward building brand image and creating prestigious experiences for elite donors. They were the first PAOs to develop philanthropy but are often stuck in a traditional concept of “fundraising” as social exchange between the agency (cultural organization) expressing a need, and the prospect (the donor). The donor gives in exchange for tangible benefits like public recognition or intangible benefits like self-actualization or personal satisfaction (Radbourne & Watkins, 2015). Faltering philanthropic income is not unusual in PAOs with low donor engagement and low-level philanthropic roles. Challenges in raising philanthropic income remain for those PAOs which do not have a supportive organizational structure.

*We now have a board member who has fundraising experience, which is fantastic! It helps to communicate at higher levels [of the organization] what we do, the energy our philanthropy strategy requires. [Board directors used to] think you just sit here and [that] the checks come in*. (Jane, Development manager, Theater Company)

Engagement strategies are not proficiently developed. Their values are linked to the prestige of belonging to a closed group of donors. Their long story in the fundraising journey has allowed them to create a foundation or fund to maximize philanthropic revenue for the future.

The results of their strategy in terms of operating performance do not necessarily reflect their organizational effort, as the philanthropy behaviors and donors have evolved over time, requiring PAOs to be more caring for the community and more oriented to engaging with donors. Indeed, they obtained higher philanthropic revenue in the early 2000s and have experienced a slow downward trend in the last decade. PAOs have a high percentage of self-generated income (with an average of 60% of the total revenue); the public grants impact only marginally on their revenue stream (about 24%); and shows more stability in targeting philanthropic income, although slightly decreasing over time within total revenue. In 2018, an average of 9.6% of revenue came from private donations, whereas they registered the highest level of philanthropy in the early 2000s (about 7%); a slight downward trend is thus evident, with the exception of the last two years. This group started with the highest percentage of donations in 2001 (average of 7%; 46% higher compared to other PAOs), but they had only a slight decrease over the years, or they have not increased this revenue stream compared to their initial achievement. This illustrated how philanthropy, and donor requests are changing over time.

### Follower

These PAOs are unstructured with philanthropic activity a prerogative of a single board member or external entity (e.g., through an external foundation or group of friends) without shared strategic direction with the organization. The philanthropic role of the PAOs whose philanthropic income trend over time is stagnant remains unfilled or is shared with other roles. Their organizational structure may undergo deep change, and the effect will only show in the long term. The main challenge that these PAOs experience in strategy implementation is the lack of professional skills in fundraising and priority in fundraising from a strategic perspective. Such PAOs need more effective communication and accountability for donations and there might have been a culture of asking for donations and communicating their values in a piecemeal fashion. The financial results of these organizations show they are strongly supported by government, with philanthropic income and partnerships marginal in their revenue stream. They are recipients of public funds who expect the status quo to continue and show stagnation in philanthropic income over time. The four PAOs show both the highest levels of public grants (average of 50%, with peak of 70%) and the lowest percentage of donations (less than 5%) over the years. They started with philanthropic donations being almost absent (2.6% in 2001), and even where donations increased, they did not exceed 5% (with the exception of 2018). Low revenue from donations was consistent over time, with no increase in philanthropy. These PAOs recorded the lowest percentage of self-generated income (on average 32.7% in 2018; with the lowest increase in the period + 2%). PAOs in this group are illustrative of organizations which are strongly supported by government, with philanthropic income and partnerships marginal in their revenue stream, and possibly showing the crowding-out effect.

The results indicate that different philanthropic traditions exist in PAOs and also that different types of structure and engagement impact philanthropic income. Neoliberal tensions can in fact arise in PAOs when structural needs are ignored and individual responsibility is assumed to be paramount (Alexander, 2018). Donor engagement is shown in the data as increasingly important as a means of increasing philanthropic income. In sum, our analysis shows that differences in resource dependency do not reflect location, size, or age of PAOs. PAOs in large cities have not necessarily made the most of their opportunities, suggesting that geographical location and access to a wider donor base are not necessarily predictors for success.

## Conclusion

The study contributes to management literature on philanthropy for arts organizations, for which raising funds from philanthropy is important for financial sustainability. We focus on PAOs where raising funds is a means to ensure sustainability rather than the main mission, as it is for organizations such as trusts, foundations (Cobb, 2002) and voluntary associations (Eikenberry, 2007). For PAOs, the core mission is to deliver high value cultural products and make them accessible to the widest possible audiences. However, this is against a background of rising neoliberalism which creates pressures and complexities in handling different types of resource acquisition, and the introduction of new models of management and governance. We offer insights into how organizations deploy and develop resources to realize artistic achievement through philanthropic income (Labaronne, 2019).

Evidence from our study shows the continuing vulnerability of some PAOs where philanthropic income has not met expectations. It supports findings of earlier studies of arts organizations in the US (e.g., Yermack, 2017); and findings on the importance of the internal structure for philanthropic success (Pierce, 2000), particularly boards which function as resource catalysts and directly impact organizational performance (Romero-Merino & García-Rodriguez, 2016). Clearly, different traditions exist in PAOs, and some organizations have managed to transform the pattern of resource dependency (Radbourne & Watkins, 2015), and also, at their best, provide space to engage donors and to use organizational resources to respond to changing philanthropic needs.

Our data support the view that both organizational structure and broad philanthropic engagement are positively linked to philanthropic income. In the period under study, the increasing dependence on external financial resources, due to public funding cuts, has forced major PAOs to innovate (Alexander, 2018; Mintzberg, 1992), and search for resources to boost donations. The factor which determines the effectiveness of a PAO in managing its capacity to attract donations is the position of philanthropy management within its internal structure (Yermack, 2017). This finding reflects those of other studies in the field, which suggest new donors are more willing to support specific causes for which they contribute as a “real community”, rather than just supporting specific PAOs (Gorczyca & Hartman, 2017; Achieve, 20). It partially explains why some PAOs which were early adopters in the field of philanthropy are now experiencing a downward trend.

In order to answer our research questions, we combined insights from two resource dependence theories which anchored our inquiry (e.g., Sandhu, 2013; Shaw et al., 2018). Combining two theoretical viewpoints provides lenses at different levels, making it possible to interpret complex motivations that trigger differing responses to resource dependence. RBV has been used as an alternate explanation for performance differences between firms in the strategic management literature (Akingbola, 2013). Although different studies have underlined how important it is to have resources in terms of board members or organization units acting as gatekeepers in the acquisition of resources (Jain & Zaman, 2020), it has also been found that excessive formalization and centralization (Sandhu & Kulik, 2019) of philanthropic structure can cause stagnation with an inability to engage externally.

The present research identifies three requirements for philanthropy to function effectively in a PAO. There is a need for: (i) balance between external and internal resources as a matter of managerial judgement. This clearly requires will and skill to be honed over time; (ii) a formalized, centralized, embedded structure underpinned by whole organization commitment to philanthropic resources, and a commitment to change where necessary; (iii) engagement with donors through effective and efficient interplay of external and internal resources in order to obtain philanthropic income. Engagement is based on interactive experience and iterative processes, and resultant mutually beneficial outcomes (Payne et al., 2008). In the case of PAOs, philanthropy provides resources that are critical for organizational survival (Pfeffer & Salancik, 2003), strengthening the mission of the organization to engage with the public and ensure different types of engagement. Our study thus extends understanding of the interplay of resources with donor engagement and organizational structure in PAOs, showing how all three factors shape and are shaped to achieve higher levels of philanthropic income through resource integration.

## Limitations and future research

We recognize the limitations of this study. First, our study is based in one country with a common law tradition, together with the United Kingdom and the United States. The 12 cases illustrate important ways in which philanthropic income is boosted through organizational structure in major PAOs, and how different organizational structures influence philanthropic income, but clearly, such a small number cannot provide conclusive evidence. However, we enriched the study with longitudinal analysis, plus interviews and content analysis, in order to provide an anchor for conclusions over time in relation to trends. We also recognize that considering only structure as an example of a resource in the RBV is a limitation. It is however true that internal resources are expressed not only in the organizational structure but also by other dynamics which play an important role in raising funds, not captured only in the formal division of labor (e.g., networks, leadership). Philanthropy has been studied mainly from a traditional management and marketing perspective, and this study makes the contribution by studying it from a governance and management perspective, integrating two perspectives, RDT and RBV. However, the world of philanthropy is changing, especially after COVID-19, and further studies with different perspectives are required for a field which is not easily dealt with in traditional management research. A useful further perspective could be financial business modelling.

Our study, set in Australia, has the limitation of being a single-country study. There is the need to extend research outside Commonwealth countries. Our findings are however relevant to other countries seeking to grow philanthropy in the arts and in the non-profit sector. Many countries are doing so, in an uncertain world where declining government income post-pandemic will accelerate global change (e.g., see McGrath & Legous, 2017; Toepler, 2018).

Questions that emerge for future research include: what changes will emerge for philanthropy in the next decade in a disrupted global field? How will philanthropy in the arts, and more broadly in the non-profit sector, respond to a more competitive marketplace with a neoliberal thrust? It remains for future researchers to take the study forward, and perhaps extend the research design to other types of arts organizations in Australia or other countries.

## Electronic supplementary material

Below is the link to the electronic supplementary material.

Supplementary Material 1

## Data Availability

Not applicable.
