# Supplementary material for: Philanthropy patterns in major Australian performing arts organizations
Source: J Manag Gov. 2023 Jan 12:1–30. Online ahead of print. doi: 10.1007/s10997-022-09657-2 (PMC9835031; doi:10.1007/s10997-022-09657-2)
Supplement: Supplementary file 1 — Supplementary Material 1 [file 10997_2022_9657_MOESM1_ESM.docx]

# APPENDIX 1. Philanthropy Income of PAOs in A$ (2000 – 2018)

| **Year** | **WAB** | **MTC** | **OA** | **QB** | **TAB** | **QTC** | **STCSA** | **SOSA** | **STC** | **OQ** | **BDT** | **BSTC** |
| --- | --- | --- | --- | --- | --- | --- | --- | --- | --- | --- | --- | --- |
| 2000 | 81,997 | 226,889 | 3,832,510 | 64,694 | 1,648,220 | 51,000 | 112,000 | 80,000 | 0 | 66,964 | 110,853 | 177,702 |
| 2001 | 107,730 | 467,038 | 3,641,901 | 44,077 | 1,814,589 | 85,000 | 155,000 | 132,000 | 0 | 66,964 | 101,947 | 125,535 |
| 2002 | 94,785 | 230,154 | 4,444,391 | 53,448 | 2,288,285 | 49,000 | 171,500 | 635,000 | 0 | 61,511 | 125,582 | 84,440 |
| 2003 | 121,985 | 242,836 | 3,740,209 | 75,091 | 1,196,521 | 41,051 | 254,000 | 88,000 | 3,209,913 | 73,997 | 65,405 | 167,317 |
| 2004 | 135,498 | 308,721 | 3,581,358 | 54,066 | 2,236,947 | 43,818 | 267,000 | 104,000 | 2,583,122 | 71,236 | 73,350 | 239,692 |
| 2005 | 153,443 | 302,681 | 2,891,025 | 101,662 | 4,206,802 | 35,977 | 212,000 | 229,000 | 2,642,014 | 93,750 | 87,864 | 87,263 |
| 2006 | 119,483 | 411,235 | 3,259,532 | 110,673 | 2,497,505 | 41,858 | 194,000 | 79,000 | 2,776,337 | 130,195 | 113,139 | 32,865 |
| 2007 | 96,175 | 443,485 | 3,300,413 | 100,059 | 2,557,881 | 40,935 | 255,500 | 172,000 | 2,865,337 | 164,108 | 78,674 | 37,986 |
| 2008 | 346,356 | 419,446 | 2,897,797 | 85,941 | 2,975,786 | 71,000 | 234,000 | 197,000 | 4,301,194 | 162,469 | 135,355 | 123,588 |
| 2009 | 237,132 | 448,887 | 3,067,191 | 115,423 | 3,116,479 | 144,000 | 174,000 | 179,000 | 3,840,342 | 311,393 | 124,315 | 97,820 |
| 2010 | 195,141 | 544,420 | 2,958,931 | 130,620 | 5,432,192 | 126,000 | 201,000 | 145,000 | 3,353,872 | 205,720 | 97,968 | 249,531 |
| 2011 | 3,513,476 | 588,905 | 3,462,772 | 160,705 | 5,691,113 | 280,000 | 172,000 | 146,000 | 1,751,998 | 165,644 | 440,584 | 1,571,256 |
| 2012 | 2,139,238 | 527,789 | 5,130,083 | 650,392 | 7,259,885 | 144,000 | 153,000 | 153,000 | 2,539,944 | 250,494 | 452,527 | 381,959 |
| 2013 | 608,393 | 606,974 | 7,660,301 | 1,702,888 | 7,250,182 | 191,000 | 222,000 | 134,000 | 2,691,612 | 438,656 | 531,512 | 420,296 |
| 2014 | 483,988 | 757,698 | 4,739,114 | 1,511,182 | 9,427,321 | 275,000 | 304,500 | 193,000 | 3,102,312 | 539,093 | 933,770 | 510,507 |
| 2015 | 881,427 | 1,887,927 | 4,743,230 | 3,717,396 | 5,389,624 | 462,000 | 289,500 | 105,000 | 2,776,806 | 290,886 | 1,053,462 | 447,501 |
| 2016 | 953,937 | 2,528,021 | 6,938,234 | 3,331,675 | 7,625,170 | 451,000 | 339,500 | 156,000 | 3,345,937 | 348,064 | 1,269,565 | 603,189 |
| 2017 | 1,248,264 | 3,782,340 | 5,951,114 | 5,374,393 | 10,454,589 | 714,000 | 466,500 | 164,000 | 2,438,808 | 260,140 | 1,333,128 | 955,635 |
| 2018 | 1,316,774 | 2,196,526 | 10,460,600 | 4,805,315 | 15,773,131 | 1,697,000 | 277,500 | 142,000 | 4,323,377 | 422,944 | 1,759,210 | 955,635 |
